# Polyphosphate Kinase from *Burkholderia cenocepacia*, One Enzyme Catalyzing a Two-Step Cascade Reaction to Synthesize ATP from AMP

**DOI:** 10.3390/ijms252312995

**Published:** 2024-12-03

**Authors:** Dianelis T. Monterrey, Leire Azcona, Julia Revuelta, Israel Sánchez-Moreno, Eduardo García-Junceda

**Affiliations:** Department of Bio-Organic Chemistry, Instituto de Química Orgánica General, CSIC (IQOG-CSIC), Juan de la Cierva 3, 28006 Madrid, Spain; leireazcona.sa@gmail.com (L.A.); julia.revuelta@iqog.csic.es (J.R.); israel.sanchez@csic.es (I.S.-M.)

**Keywords:** biocatalysis, ATP regeneration, polyphosphate, polyphosphate kinase, cofactor recycling, cascade reactions, *Burkholderia cenocepacia*

## Abstract

This study characterizes a novel polyphosphate kinase from *Burkholderia cenocepacia* (*Bc*PPK2-III), an enzyme with potential applications in ATP regeneration processes. Bioinformatic and structural analyses confirmed the presence of conserved motifs characteristic of PPK2 enzymes, including Walker A and B motifs, and the subclass-specific residue E137. Molecular docking simulations showed AMP had the highest binding affinity (−7.0 kcal/mol), followed by ADP (−6.5 kcal/mol), with ATP having the lowest affinity (−6.3 kcal/mol). It was overexpressed in *Escherichia coli*, after purification enzymatic activity assays revealed that *Bc*PPK2-III needed divalent cations (Mg^2^⁺, Mn^2^⁺, Co^2^⁺) as cofactors to be active. Functional assays revealed its ability to synthesize ATP from AMP through a stepwise phosphorylation mechanism, forming ADP as an intermediate, achieving 70% ATP conversion (TTN 4354.7) after 24 h. Kinetic studies indicated cooperative behavior and substrate preference, with AMP phosphorylation to ADP being the most efficient step. The enzyme demonstrated high thermostability (T_50_ = 62 °C) and a broad pH stability range (pH 6.0–9.0), making it suitable for diverse biocatalytic applications. The study highlights *Bc*PPK2-III as a robust and versatile candidate for cost-effective ATP regeneration, offering advantages in industrial processes requiring stoichiometric amounts of ATP.

## 1. Introduction

Adenosine 5′-triphosphate (ATP) is one of the most important molecules in living systems and probably the foremost phosphate compound of all. Normally known as the universal energy “currency” or “store” of the cell [1], it is also used as a building block for nucleic acid synthesis and is involved in the synthesis of key phosphorus-containing intermediates for biosyntheses and cofactors such as flavin adenine dinucleotide (FAD), nicotinamide adenine dinucleotide (NAD^+^), *S*-adenosylmethionine (SAM), and 3′-phosphoadenosine 5′-phosphosulfate (PAPS) [2,3,4,5]. ATP is a crucial molecule for many biocatalytic processes; however, its industrial application is limited by the requirement for stoichiometric amounts of this costly cofactor. With a price of approximately USD 35 per gram, depending on the supplier and grade, the high cost of ATP poses a significant challenge to the economic viability of large-scale ATP-dependent biocatalytic processes. ATP recycling systems can provide higher yields in these reactions by shifting the reaction equilibrium toward the desired product [6]. Other benefits of ATP recycling systems include reduced product inhibition by decreasing the concentration of the side product ADP, while keeping the ATP concentration high to facilitate phosphorylation reactions, simplified product purification, and cost savings by reducing the required amount of this expensive substrate [7].

**Figure 1:**
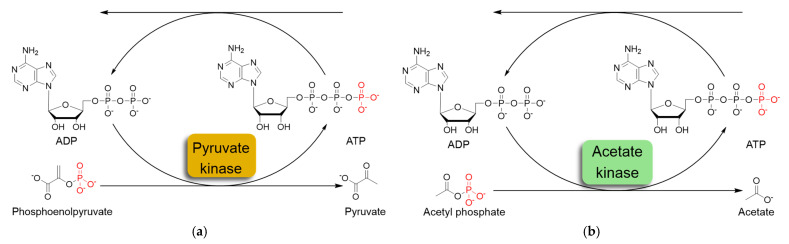
Schematic representation of in situ ATP regeneration systems using pyruvate kinase (**a**) and acetate kinase (**b**).

The most commonly used ATP regeneration systems from ADP are based on the use of phosphoenolpyruvate as the phosphate donor (Figure 1a) in a coupled reaction catalyzed by pyruvate kinase (PK) and on the use of acetylphosphate (Figure 1b) coupled with acetate kinase (AK) [8].

**Figure 2:**
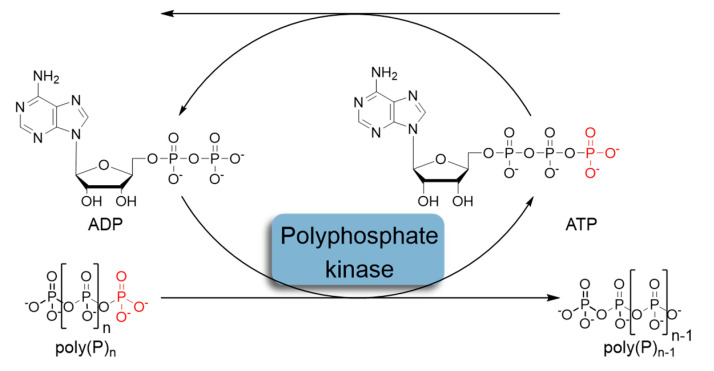
Schematic representation of in situ ATP regeneration systems using polyphosphate kinase and inorganic polyphosphate as the phosphoryl donor.

In recent decades, the regeneration system based on the use of polyphosphate kinases (PPK) that catalyze the reversible transfer of γ-phosphate from ATP to inorganic polyphosphate (polyP) has attracted considerable attention (Figure 2) [9,10]. Inorganic polyphosphate is a linear polymer consisting of up to hundreds of orthophosphate (Pi) residues linked by high-energy phosphoanhydride bonds [11,12,13].

Regeneration systems based on the use of PPKs have several advantages that make them very attractive in terms of their application in biocatalysis. First and foremost, polyP is a low-cost phosphate group donor. A large amount of polyP is regularly produced as sodium hexametaphosphate (about 13–18 residues) for industrial purposes such as food additives, which also makes polyP inexpensive compared to other phosphoryl group donors. To get an idea of the magnitude of savings from using polyP as a phosphate donor, consider that a commercial form of polyP costing about USD 20/kg can provide ATP equivalents that would cost more than USD 35,000/kg, and that other phosphate donors commonly used in ATP regeneration, such as phosphoenolpyruvate (PEP), are even more expensive than ATP (USD 264,000/kg) [14,15,16]. The other major advantage of this system is that there are PPKs that allow regeneration of ATP from AMP.

**Figure 3:**
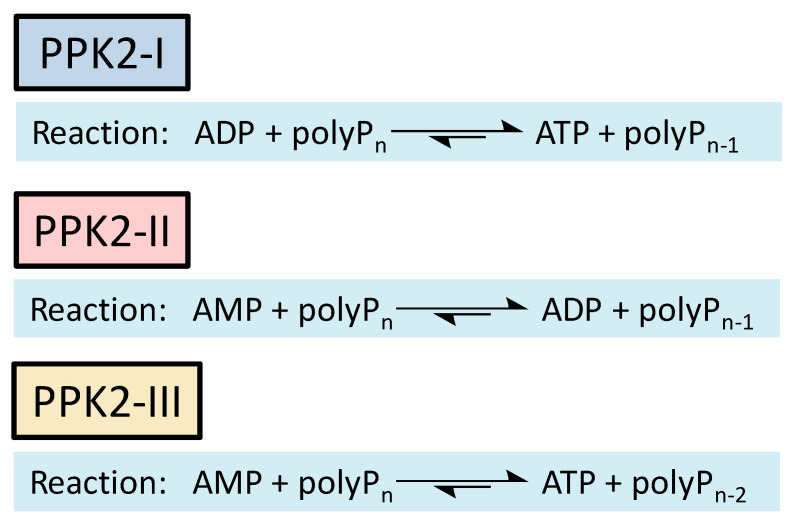
Reactions catalyzed by the different PPK family 2 (PPK2) subclasses.

Methods to regenerate ATP from AMP or adenosine are not well established, although they have great potential for biocatalysis [9,17]. There are several cases where the regeneration of ATP from AMP based on low-cost polyphosphate as the sole energy source would be of great value, such as the regeneration of acyl-CoA with integrated ATP regeneration [18] or the in situ synthesis of PAPS in reactions catalyzed by sulfotransferases [19]. PAPS is synthesized from sulfate and two ATP molecules through ATP sulfurylase, forming adenosine 5′-phosphosulfate (APS), and APS kinase, which phosphorylates APS to PAPS, consuming a second ATP molecule [20,21]. Polyphosphate kinase enzymes play an important role in regulating intracellular nucleotide concentrations in vivo by participating in the reversible transfer of phosphate groups between nucleotides and polyP [22]. To date, two major families of highly conserved PPKs have been characterized in bacteria that are neither sequentially nor structurally related. Polyphosphate kinase 1 (PPK1) is responsible for the synthesis of polyP using ATP as a donor of phosphate groups, while polyphosphate kinase 2 (PPK2) enzymes are responsible for its degradation [22]. PPK2 can be divided into three subclasses (I, II, and III) depending on the reaction they catalyze, as shown in Figure 3. They all use polyP as a phosphate group donor to catalyze the phosphorylation of the various nucleotides (AMP, ADP and ATP). The substrate preference of PPK2-II is AMP from which they can synthesize ADP. The substrate preference of PPK2-I and PPK2-III is ADP and AMP, respectively [22,23].

Given the interest that class III of PPK2 represents from a biocatalysis point of view, we consider it necessary to identify and describe new members of this enzyme family in order to increase the tools available for the development of efficient ATP regeneration processes. Although several gene sequences have been found that could encode PPK2-III enzymes, most of them are putatively assigned, i.e., they lack experimental verification. In this regard, Motomura et al. (2014) [24] constructed an unrooted phylogenetic tree of PPK2 using amino acid sequences from 17 characterized and 192 putative PPK2 homologs. This phylogenetic analysis revealed 40 sequences belonging to the class III subfamily. However, only the sequence from the bacterium *Meiothermus ruber* was amplified and expressed to characterize the biochemical properties of the PPK2-III enzymes.

*Burkholderia cenocepacia* is a Gram-negative bacterium and belongs to the *Burkholderia cepacia* complex (Bcc). Although common in the environment, it can also cause disease in plants and act as an opportunistic pathogen that frequently infects patients with cystic fibrosis and chronic granulomatous diseases. Studies have shown that *B. cepacia* isolates from various sources accumulate higher levels of intracellular polyphosphate (polyP) and increase the uptake of inorganic phosphate when grown in acidic environments [25]. The importance of polyP metabolism in this bacterium suggests that *B. cenocepacia* could be an interesting source for the overexpression and characterization of a new PPK2-III with interesting properties for its application in biocatalysis.

Herein, in this work, we addressed the characterization of the putative polyphosphate kinase from *Burkholderia cenocepacia*. A study of its secondary and tertiary structure was performed using theoretical structural analysis and 3D modeling. The enzyme was overexpressed in *E. coli*, and after its purification, its enzymatic activity, divalent cation dependence and reaction mechanism were studied. Enzyme kinetic parameters and stability were also analyzed and discussed. In addition, we have been concerned with elucidating the mechanism of action of this enzyme, as it is controversial whether it is a stepwise sequential diphosphorylation mechanism or a pyrophosphorylation mechanism and thus occurs in a single step [23].

## 2. Results and Discussion

### 2.1. Bioinformatic Analysis and Structural Modeling of BcPPK2-III

#### 2.1.1. Sequence Analysis of the Putative PPK2-III from *Burkholderia cenocepacia*

PPK2 belongs to the P-loop kinases. This protein fold is by far the most common among enzymes that bind or hydrolyze nucleoside triphosphates (NTPs) and is characterized by the presence of two conserved sequence motifs (Walker A and Walker B) and a lid module (RxxxxxxPxxxxxxxD). The Walker A (DxxGK) and Walker B (RSxY) motifs are conserved in all PPK2 subclasses, whereas the amino acid following the Walker B motif appears to be subclass-specific. These amino acids are asparagine (subclass I), glycine (subclass II), and glutamic acid (subclass III) [24,26,27].

Using as query the sequence of the PPK2-III enzyme from *Meiothermus ruber* (Mrub_2488), a sequence alignment of the putative PPK2-III from *B. cenocepacia* was performed using the BLAST^®^ program (Figure S1) [28,29]. The sequence analysis shows 51% identity, and the *E*-value, indicating the probability of alignment occurring by chance, was 10^−95^. Structural comparison showed excellent agreement between the two proteins, with a root mean square deviation (RMSD) of 1.14 Å and a template modeling score (TM-score) of 0.97, confirming a high degree of structural similarity. Conserved motifs characteristic of PPK2 enzymes, including Walker A and B motifs and the cap module, were identified in the *B. cenocepacia* sequence (Figure S1). Additionally, the sequence contains glutamine (Q74) preceding the Walker A motif and glutamic acid (E137) downstream of the Walker B motif, the latter being the class III signature residue of PPK2 and contributing to the binding of the adenine base [27]. Mutational studies have shown that E137 plays a critical role in phosphorylation activity for AMP, ADP, and GDP, although it does not solely determine substrate specificity [30].

The sequence of *Bc*PPK2-III was also analyzed for possible transmembrane regions using the protein topology prediction method, TMHMM, based on a hidden Markov model [31]. As no such zone was found, we can assume that the protein is free in the cytosol, which increases the chances that it will be expressed in the soluble form.

#### 2.1.2. Molecular Modeling of *Bc*PPK2-III

**Figure 4:**
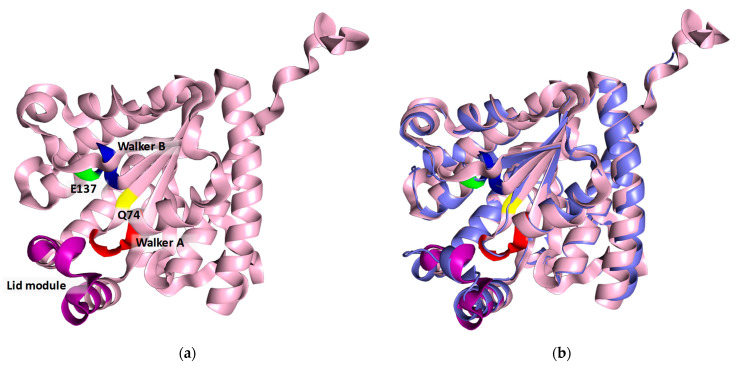
(**a**) Model of the 3D structure of the recombinant protein *Bc*PPK2-III. The conserved motifs are shown in colors: Walker A is shown in red, Walker B in blue and the lid module in violet. The conserved amino acids Q74 and E137 are displayed in yellow and green, respectively. (**b**) Structural alignment of *Bc*PPK2-III (light pink) with *Mrub*PPK2-III (PDB: 5LD1) (slate gray).

The 3D structure of the protein was modeled using the ColabFold v1.5.5 open-source software [32,33] completed with 3D structure modeling. The high Model Confidence and Sequence coverage (Figure S2a and b, respectively) indicate the good quality of the predicted enzyme structure. The vast majority of the structure has been predicted with a very high (>90%) degree of confidence (Figure S2a). The obtained results were visualized using PyMOL. The three conserved motifs (Walker A motif, Walker B motif, and lid module) are highlighted in red, blue, and green, respectively (Figure 4). The spatial position and proximity of the three motifs supports the hypothesis that the active center is located below the lid module, between motifs A and B [24,27].

#### 2.1.3. Modeling of the Enzyme–Substrate Complexes

Molecular docking is a helpful tool in predicting the affinity and biding mode of a native molecule in the enzyme active site. In order to harness a more complete understanding of *Bc*PPK2-III and the molecular foundation of interaction and conformation of the three main compounds within the binding pose, a docking study was performed using Maestro Schrödinger’s Glide module. The results of the molecular docking are summarized in Table 1, which lists the conformer of each substrate that yields the best docking score, as well as the residues of the active site of the enzyme involved in the interaction with the substrates.

The docking scores indicated that AMP had the highest binding affinity with a score of −7.0 kcal/mol, followed by ADP with a score of −6.5 kcal/mol, while ATP showed a relatively lower affinity with a score of −6.3 kcal/mol. The Glide Emodel results provided further insights into the stability and quality of the binding poses. ATP exhibited the most favorable Emodel score of −136.7 considering the lower docking score, suggesting a highly stable interaction within the active site. This suggests that ATP binds with more internal strain than the other ligands. This strain could arise from conformational adjustments required to accommodate the triphosphate moiety within the active site. Such adjustments could make the binding less energetically favorable, lowering the docking score despite having strong overall stability. AMP also demonstrated a strong binding pose with an Emodel score of −100.3. ADP showed intermediate values on both parameters. These results underscore the nuanced interplay between binding affinity and pose stability, highlighting AMP as the most suitable ligand.

**Figure 5:**
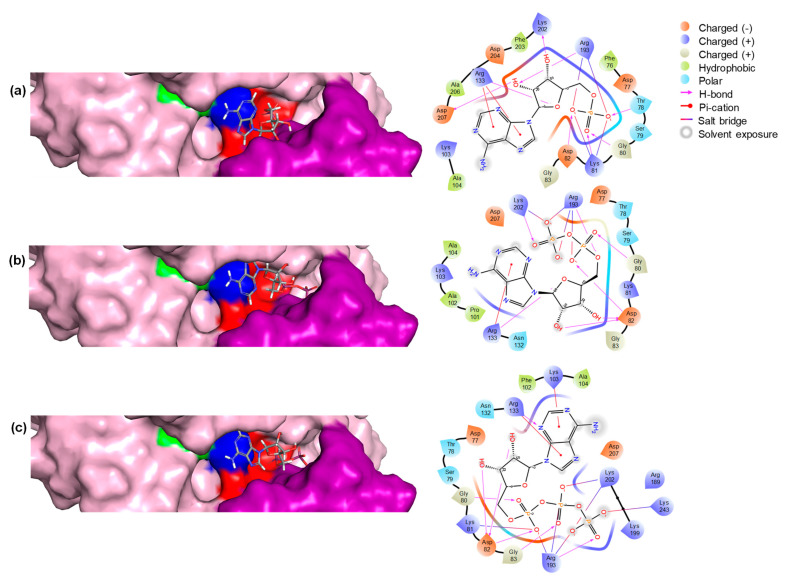
Structural representation of the enzyme with three different substrates bound in the active site. (**a**) The enzyme is bound with AMP, (**b**) ADP, and (**c**) ATP. Each panel includes a 3D structure of the enzyme–substrate complex and a 2D representation showing the residues interacting with the compounds. In the 3D structures, key residues are highlighted: Walker A motif residues in red, Walker B motif residues in blue, glutamic acid (E) in green, and the lid domain residues in violet.

The biding pose and residue interaction of the three ligands are displayed in Figure 5. The results are consistent with the expected binding of the three compounds, as has been described before [34].

The interactions between ATP, ADP, and AMP with the enzyme are mediated by several key residues that are critical for binding and catalytic activity. In particular, residues from the Walker A motif, like Asp82 and Gly80, play a critical role in anchoring the nucleotides, while Thr78 and Lys81 enhance binding stability through hydrogen bonding and electrostatic interactions. The Walker B motif residues, Arg133 and Arg193, further stabilize the interactions with the phosphate groups, enhancing binding affinity. Additionally, Lys202 from the lid motif helps maintain the structural integrity of the active site, ensuring proper positioning of the nucleotides for optimal catalytic activity. These interactions highlight the importance of these residues in facilitating nucleotide binding and enzyme function.

AlphaFold3 modeling of *Bc*PPK2-III with different divalent cations (Ca^2^⁺, Mn^2^⁺, Mg^2^⁺, and Co^2^⁺) and ligands (ATP, ADP, and AMP) revealed no significant structural differences among the predicted models. The enzyme’s overall fold and active site architecture remained consistent regardless of the specific cation or ligand present. Detailed visualizations of the modeled structures are presented in Figure S3.

### 2.2. Heterologous Overexpression, Purification, and Identification of BcPPK2-III

Expression of the recombinant protein was induced with 0.5 mM IPTG at an OD600 nm of 0.5–0.6. The expression and purification of the *Bc*PPK2-III enzyme was analyzed by SDS-PAGE (Figure S4). The system yielded a high degree of overexpression of the target enzyme, with 71 mg of pure protein per liter of culture. The CFE (Figure S4, lane 1) showed a majority band corresponding to the recombinant protein given its theoretical size (32 kDa). Densitometric analysis revealed that the recombinant protein accounted for 60% of the total soluble proteins in the CFE. The analysis of the IMAC purification process of the recombinant protein shows that the recombinant protein binds very specifically to the resin, as it was not detected in the flow-through after loading the CFE (Figure S4, lane 2), nor in the subsequent washes performed to remove proteins not specifically bound to the resin (Figure S4, lane 3). On the contrary, in the combined fractions obtained after elution of the recombinant protein with imidazole (Figure S4, lane 4), a single band corresponding to *Bc*PPK2-III was observed with a purity higher than 98%. Interestingly, when the resin was analyzed after elution of the recombinant protein (Figure S4, lane 5), it was found that a considerable amount of the protein remained bound to the resin with a high degree of purity (77.5%). We did not analyze whether the protein that remained bound on the resin was functional or not. In any case, our results indicate that the protein binds very strongly and selectively to the Co^2+^-IDA resin, providing an efficient method for simultaneous purification and immobilization of the recombinant enzyme.

The identity of the recombinant protein was confirmed by peptide mass fingerprinting (Figure S5). Seventeen different peptides were identified, corresponding to 68% of the protein sequence. This result confirmed that the sequence of the purified protein matched the sequence of *Bc*PPK2-III.

### 2.3. Functional Characterization of BcPPK2-III

#### 2.3.1. Metal Cofactor Dependence of *Bc*PPK2-III

**Figure 6:**
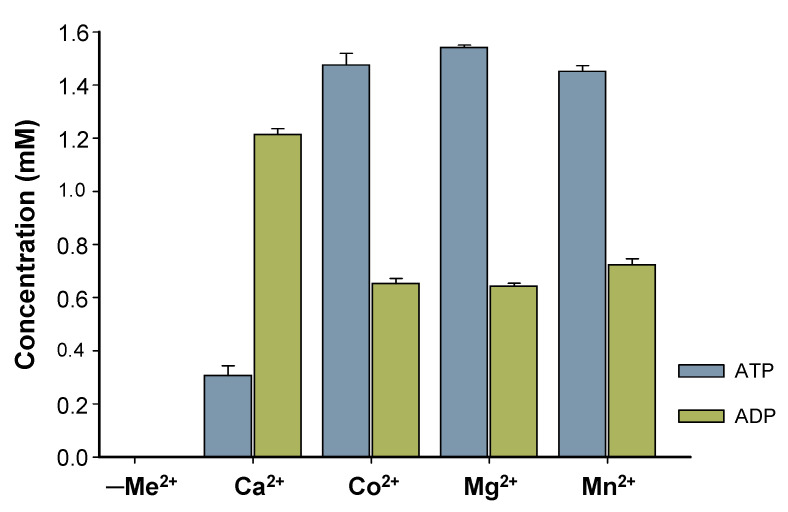
Effect of metal ions in enzyme activity. Reaction after 24 h using the same concentration of polyP_12_ (10 mM) and same concentration of divalent cation (10 mM), for Ca^2+^, Co^2+^, Mg^2+^, Mn^2+^. Concentration of produced ATP (blue) and ADP (green) is represented. A negative control (reaction mixture without cation, indicated as -Me^2+^) is included.

Characterization of *Bc*PPK2-III initially focused on the enzyme metal cofactor determination. Although the Luria–Bertani (LB) medium used for protein expression contains trace amounts of metal ions, these were effectively removed during purification via immobilized metal affinity chromatography (IMAC). During elution, imidazole displaced any metal ions bound to the protein by competing with histidine residues for metal coordination. This process ensured that the purified *Bc*PPK2-III was devoid of residual metal ions from the expression medium. Four different divalent cations (Mg^2^⁺, Mn^2^⁺, Co^2^⁺ and Ca^2^⁺) were tested. The enzymatic assays revealed no activity in the absence of added divalent cations (negative control, Figure 6). *Bc*PPK2-III shows activity across all tested cations (Figure 6) and exhibits substantial and similar activity towards the production of ATP when Co^2+^, Mg^2+^, and Mn^2+^ are used as cofactors, confirming that the enzyme’s catalytic function relies on the deliberate addition of metal ions post-purification. Interestingly, when the enzyme used Ca^2+^ as a cofactor, it exhibited distinct behavior wherein the production of ADP surpassed that of ATP. Determining this specificity is of great interest because it has been described that metal cofactor preference plays a crucial role in the efficiency of the enzyme towards ATP production. Generally PPK2 enzymes show dependence on Mn^2+^ [24] or Mg^2+^ [27]. In the cases of PPK2-III from *Meiothermus ruber* and PPK2-III from *Ralstonia eutropha,* both divalent cations were used by the enzyme to produce ATP [20,24].

This is consistent with Nocek and colleagues [30] since PPK2-III was reported to exhibit high and similar activity with three different cations, illustrating the versatility of the enzyme along with its distinct Ca^2+^-dependent activity. Further studies should be performed to explain the different behavior found with Ca^2+^, considering the role of its smaller ionic radius and the solvation shell of the cation.

In addition, it is worth mentioning that the 24 h reaction using Mn^2+^ as an enzyme cofactor resulted in a precipitate that could indicate the formation of a nanoflower-like structure [35] between the shorter polyP chains and the cation, resulting after enzyme activity (Figure S6). This could translate into lower availability of the phosphate groups, which could influence the enzymatic efficiency of the enzyme to produce ATP. In this sense, further characterization of the enzyme was carried out using Mg^2+^ as a metal cofactor.

#### 2.3.2. Reaction Mechanism and Stepwise Phosphorylation Pathway

**Figure 7:**
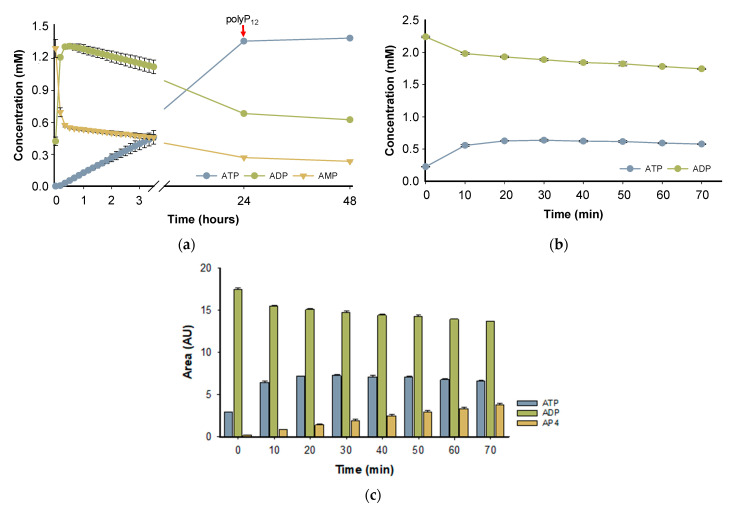
Reaction time-course, showing the concentration of AMP (yellow triangles), ADP (green circles) and ATP (blue circles). In both reactions, the starting material concentration was 2 mM, Mg^2+^ was used as divalent cation and PolyP_12_ (10 mM) as a phosphate donor, and the experiments were performed in triplicate. (**a**) The starting material of the reaction was AMP and the red arrow indicates the supplementation of the reaction with PolyP_12_ (10 mM). (**b**) The reaction was carried out with ADP as the initial substrate. (**c**) The time-course of the reaction initiated with ADP as the substrate, showing the appearance of AP4.

The PPK2-III family can perform ATP formation from AMP through two main reaction mechanisms. The most common mechanism is step-by-step phosphorylation, in such a way that starting from AMP, the enzyme catalyzes the formation of ATP with an intermediate formation of ADP. On the other hand, the direct production of ATP from AMP in the so-called pyrophosphorylation mechanism has been reported, adding two phosphate groups to AMP, without forming the intermediate ADP [24,36]. To assess the enzymatic mechanism of *Bc*PPK2-III, the progress of the reaction over 48 h was analyzed (Figure 7a). According to the time-course experiment, in the first 10 min of the reaction, the enzyme produces ADP and, in a lower rate, forms ATP, reaching a high yield (≈70%) after 24 h. PPK2-III enzymes prefer larger chains of polyP_n_ and the shortening of the phosphate donor during the reaction is a limiting factor in enzyme activity [25,32]. Subsequently, the reaction was supplemented with polyP_12_ and the analysis after 48 h did not show a significant increase in ATP yield, probably due to enzyme stability or inhibition.

To further support the hypothesis that the reaction mechanism proceeds by a stepwise phosphorylation process and that the observed decrease in ADP is not due to adenylate kinase side reactivity, a reaction was performed with ADP as the starting substrate instead of AMP. Figure 7b shows the reaction time-course using ADP as a substrate. When using ADP as a substrate, we observed the formation of ATP, which was confirmed by comparison with a standard in HPLC analysis. In addition to ATP, a third peak was detected, which we hypothesize to be adenosine tetraphosphate (AP4) based on its elution profile, UV spectra and retention time (Figure 7c and Figure S7c). However, further analysis is required for confirmation. These results are in agreement with the published literature, since it has been described that PPK2 enzymes can mediate the formation of oligophosphorylated products up to nucleoside nona-phosphates [37].

These results experimentally confirm that the *Bc*PPK2-III enzyme belongs to subclass III, as it catalyzes the stepwise phosphorylation of AMP to ADP and subsequently to ATP.

#### 2.3.3. Kinetic Analysis and Catalytic Efficiency of *Bc*PPK2-III

It is noteworthy that the PPK2-III enzyme displayed a sigmoidal curve in all three reactions studied (AMP→ATP, AMP→ADP and ADP→ATP) instead of the expected hyperbolic profile in the Michaelis–Menten kinetics (Figure S8). The nonlinear regression analysis performed on the experimental data showed a better fit to the Hill equation than to the classical Michaelis–Mentel equation, which was confirmed with directed and secondary kinetics data plotting. The fit to the Hill equation model was assessed by calculating the R^2^ of the fit, which was 0.95 for the AMP to ATP reaction, 0.89 for the AMP to ADP reaction, and 0.96 for the ADP to ATP reaction. These values are significant and reproducible as the experiments were carried out in triplicate. In addition, the Hill coefficient (n) was greater than 1 in all three cases—4.8 for the AMP to ATP reaction, 2.9 for the AMP to ADP reaction, and 3.7 for the ADP to ATP reaction—indicating positive cooperativity. Positive cooperativity is unusual in simple enzyme kinetics and suggests a structure in which interactions between sub-sites within the active center (Figure 5) facilitate the sequential progression of the reaction, as observed in enzymes with multiple phosphorylation sites or cascading active subunits. This cooperative behavior complicates the accurate calculation of kinetic constants, as the kinetic behavior of the enzyme is strongly influenced by the experimental conditions. Despite these challenges, we present reliable kinetic data for each catalytic step, with calculated apparent constants that align with previously reported values for comparable enzymes (https://www.brenda-enzymes.org/enzyme.php?ecno=2.7.4.1 (accessed on 4 November 2024)).

The catalytic efficiency of the enzyme, as assessed by total turnover number (TTN), initial turnover rate (ITR) and kinetic constants (Table 2), showed that ATP production is significantly more efficient when ADP serves as a substrate, achieving a rate approximately eight times higher (1240 M⁻¹s⁻¹) than with AMP. Additionally, the phosphorylation efficiency of AMP to ADP is more than three times higher than that of AMP to ATP, with correspondingly higher ITR values for the conversion of AMP to ADP. This result highlights the importance of substrate choice in optimizing enzyme performance.

#### 2.3.4. Enzyme Stability

**Figure 8:**
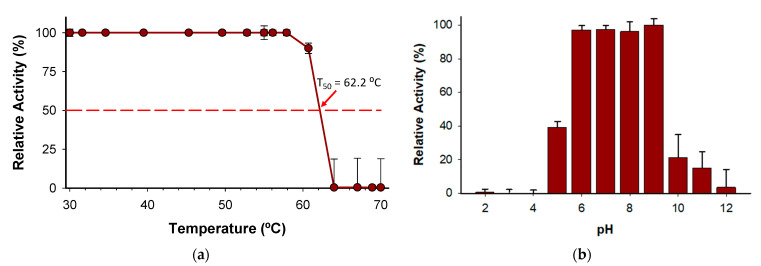
(**a**) Kinetic thermostability (T_50_) of *Bc*PPK2-III, (**b**) pH profile. Values are presented as the mean ± standard deviation of three independent experiments.

The T_50_ (the temperature at which the enzyme retains 50% of its activity after a ten-minute incubation) was determined to be 62 °C (Figure 8a). Notably, the enzyme holds 100% activity from 30 to 58 °C, which indicates a high degree of thermostability considering other enzymes described belonging to the same family [24]. In addition, the stability of the enzyme has been evidenced by pH profile experiments (Figure 8b) since the enzyme retains more than 97% of activity from pH 6.0 to 9.0. The wide range of pH activity exhibited by the novel *Bc*PPK2-III is a distinctive attribute that enhances its versatility to be included in several systems as an ATP regeneration module. Enzymes from this family have been described to show activity across a wide pH range, including neutral, acidic and alkaline conditions [36,38].

## 3. Materials and Methods

### 3.1. Materials and General Procedures

Synthesis of the gene *ppk2-III* from *Burkholderia cenocepacia*, optimization of the nucleotide sequence, and cloning into the plasmid pET-28b(+) were performed by GenScript (Piscataway, NJ, USA). Peptide mass fingerprinting was performed at the Proteomics Unit of the Complutense University of Madrid. *Escherichia coli* strain BL21 (DE3) was obtained from Promega Biotech Ibérica S.L. (Madrid, Spain). The GenEluteTM Plasmid Miniprep Kit from Sigma-Aldrich (Darmstadt, Germany) was used to purify the plasmid. Promega 1kb DNA Step Ladder Molecular Weight Marker and restriction enzymes were purchased from ThermoFisher Scientific Inc. (Waltham, MA, USA). Kanamycin, DNase I, and other reagents were purchased from Sigma-Aldrich (Darmstadt, Germany). Isopropyl-β-d-thiogalactopyranoside (IPTG) was purchased from Applichem GmBH (Darmstadt, Germany). The acrylamide/bis-acrylamide 30% (29:1) solution used for protein analysis by SDS-PAGE was purchased from Bio-Rad (Hercules, CA, USA). The Low Molecular Weight Calibration Kit from GE Healthcare was used. SDS-PAGE gels were run on a MiniProtean^®^ Tetracell cuvette from Bio-Rad (Hercules, CA, USA). Agarose gels were run in a RunOne™ Electrophoresis Cell cuvette from EmbiTec (San Diego, CA, USA) using SYBR Safe from ThermoFisher Scientific (Waltham, MA, USA). Analysis of the gels was performed using a Gene Flash Bio Imaging Photodocumenter from Syngene LTD (Bengaluru, Karnataka). Iminodiacetic acid agarose (IDA-agarose) was purchased from Agarose Bead Technologies (Miami, FL, USA). Solvents were of analytical grade. DNA manipulation was performed according to standard procedures [39].

### 3.2. Bioinformatic and Computational Analysis

#### 3.2.1. Sequence Analysis and Structural Modeling of *Bc*PPK2-III

Secondary structural motifs of the protein were identified by the theoretical structural analysis performed in the PSIPRED Protein Analysis Workbench [40]. Three-dimensional structure modeling without a template was performed using the ColabFold open-source software [34,35]. In addition, the structure of *Bc*PPK2-III was modeled using AlphaFold 3, a state-of-the-art deep learning algorithm for protein structure prediction. The modeling was conducted to explore potential interactions between the enzyme and various divalent cations (Ca^2^⁺, Mn^2^⁺, Mg^2^⁺, and Co^2^⁺) as well as ligands (ATP, ADP, and AMP). The input sequence of *Bc*PPK2-III was used without additional structural templates or constraints, and the resulting models were analyzed for cation- and ligand-binding configurations.

Pairwise structural alignment was performed using the RCSB PDB tool to compare the 3D structure of *Bc*PPK2-III with *Mrub*PPK2-III (PDB: 5LD1), taking into account RMSD values and TM-score [41].

#### 3.2.2. Molecular Docking Modeling of the Enzyme–Substrate Complexes

The docking study was conducted using Maestro (version 13.2.128) for molecular modeling and visualization and MMshare (version 5.8.128, Release 2022-2) for molecular mechanics. The protein structure was prepared using Maestro. Ligand structures were sketched using Chemdraw and subsequently prepared using Maestro’s ligand preparation tools. This preparation involved generating 3D coordinates, optimizing the ligand geometry, and assigning appropriate force field parameters. The ligand structures were energy minimized using the OPLS3e force field within Maestro to ensure accurate docking results. Docking simulations were performed using the Glide module within Maestro. The protein’s active site was defined by setting a grid box around the binding pocket, and the ligands were docked into this grid to explore potential binding modes. Default Glide settings, including standard precision (SP) mode, were used for the docking simulations. Each ligand was docked in multiple poses, and the results were analyzed to identify binding modes with favorable docking scores. The top-ranked binding poses were selected based on their docking scores and interactions with the active site residues. Binding interactions and the best docking poses were visualized and analyzed using Maestro’s visualization tools to assess the quality of ligand binding.

### 3.3. Expression and Purification of BcPPK2-III

#### 3.3.1. Cloning of *ppk2-III* Gene

The *ppk2-III* gene from *B. cenocepacia* was synthesized and optimized for expression in *E. coli*. This significantly improved the codon adaptation index (CAI) from 0.68 to 0.97. Considering that a CAI value of 1 occurs when the sequence is optimal for expression in the specific organism, the performed optimization significantly improves the chances of correct expression of the *ppk2-III* gene in *E. coli*. The percentage of guanines (G) and cytosines (C) could be decreased by 8%, which could significantly increase the stability of DNA and the secondary structure of the mRNA. Cloning of the optimized *ppk2-III* gene was performed in the expression plasmid pET-28b(+), which allows induction of protein overexpression with isopropyl-β-d-1-thiogalactopyranoside (IPTG). The protein is expressed fused to a 6-His tag at the N-terminus, allowing its one-step purification by ion metal affinity chromatography (IMAC) from cell-free extract (CFE). The plasmid, designated pET-28b(+)-*ppk2-III*, was then transformed into competent *E. coli* BL21 (DE3) cells. Restriction analysis of the plasmid after its purification revealed two bands (Figure S9): the band corresponding to the plasmid pET-28-b(+), with a size of 5368 bp, and a band of 279 bp corresponding to the expected size of the *ppk2-III* gene.

#### 3.3.2. Heterologous Expression and Purification of Recombinant *Bc*PPK2-III

For expression of the *Bc*PPK2-III protein, preinocula of recombinant colonies were prepared in 5 mL LB medium containing 5 μL kanamycin (50 μg/mL) and incubated overnight at 37 °C with shaking (160 rpm). The culture was extended and incubated under the same conditions until the exponential phase (OD_600nm_ = 0.5–0.7) was reached. At this point, protein expression was induced with IPTG at a final concentration of 1 mM. Orbital agitation was kept constant and the temperature was lowered to 30 °C to avoid the formation of inclusion bodies. The culture was then centrifuged at 4000 rpm for 20 min to recover the cells. The pellet was resuspended in Na_2_HPO_4_ buffer (50 mM, 300 mM NaCl, pH = 8.0). Cell disruption was performed by sonication (70% amplitude, 10 s pulse and 15 s pause). The lysed cells were separated by centrifugation of the mixture at 8000 rpm for 20 min. The supernatant obtained was treated with DNaseI (10 µg/mL cells) and MgCl_2_ (0.95 µg/mL cells) for 20 min on ice. Streptomycin (1% weight/volume) was then added and allowed to act for an additional 20 min. Finally, the cell-free extract (CFE) was obtained by centrifugation, which, after aliquoting, was frozen with liquid NO_2_ and stored at −80 °C.

Purification by divalent ion affinity chromatography (IMAC) was performed using a Co^2+^-IDA–agarose column. The resin was washed with milliQ H_2_O and equilibrated with 10 mM imidazole, 50 mM Na_2_HPO_4_ and 300 mM NaCl buffer, pH = 8.0. CFE was added to the resin at a ratio of 1:1 (*v*/*v*). To remove proteins non-specifically bound to the resin, the column was washed with 2 volumes of the same buffer. The recombinant protein was then eluted with 1 volume of the same buffer containing 2.0 M imidazole. The eluates were dialyzed to remove the imidazole on 30 kDa cut-off Vivaspin centricones. Finally, the enzyme was kept in Tris-hydroxymethyl-aminomethane (Tris-HCl) buffer (50 mM pH = 8.0 at 4 °C). The protein concentration in the purification fractions was quantified by Ab_280nm_ using the theoretical size of the protein (32 kDa) and the theoretical molar extinction coefficient (ε) determined by the content of tryptophan (W), tyrosine (Y), and cysteine (C) and calculated using Equation (1):

ε = (nW × 5500) + (nY × 1490) + (nC × 125)(1)

Expression and successive purification steps were monitored by SDS-PAGE (Figure S4) at 11% in the resolving gel. Quantification of gels and protein purity was performed by densitometric analysis (GeneTools 3.07).

### 3.4. Functional Characterization and Stability Assays

#### 3.4.1. HPLC Analysis of *Bc*PPK2-III Activity

Analysis of the enzymatic activity of *Bc*PPK2-III in all cases was performed by reverse high-performance liquid chromatography (RHPLC) with an Agilent 1260 Infinity II equipped with an InfinityLab Poroshell 120 EC-18 column (150 mm × 4.6 mm, 4 μm) and photodiode array (PDA). The injections were performed by an autosampler and 3 μL of enzyme reaction was injected. The solvent system used solvent (A): 0.1% (*v*/*v*) trifluoroacetic acid (TFA) in H_2_O and solvent (B): 0.05% (*v*/*v*) TFA in CH_3_CN, with a flow rate of 1 mL min^−1^, detection at 260 nm and column temperature of 30 °C. Samples were analyzed using a gradient elution of 100% A over 4 min and of 0 to 10% of B over 4 min. Commercial standards of ATP, ADP, and AMP with retention times of 2.8 min, 3.5 min and 7.3 min, respectively, were used to identify the different nucleotides present in the reaction mixture (Figure S7).

#### 3.4.2. Enzyme Activity Assay, Metal Ion Preference and Reaction Progress

Unless stated otherwise, enzyme activity assays were performed using 100 mM Tris-HCl buffer pH = 7.0, polyP (10 mM) with a chain length of 12 units as the donor of phosphate groups, AMP or ADP as substrates with a concentration of 2 mM, and MgSO_4_ (10 mM) as the metal ion cofactor. Reactions were initiated by the addition of *Bc*PPK2-III (10 µg/mL) and maintained at 30 °C with orbital shaking (700 rpm). *Bc*PPK2-III reaction progress was monitored by RHPLC every 10 min for the first 6 h and after 24 h. Additionally, 10 mM of polyP was supplemented to the same reaction and measured after 48 h.

A study of the enzymatic activity of *Bc*PPK2-III was performed using the standard conditions and introducing various metal ions (Ca^2+^, Co^2+^, Mg^2+^ and Mn^2+^) with a final concentration of 10 mM in the reaction.

#### 3.4.3. Kinetic Constants Determination

The kinetic constants determination was performed with the following conditions: 100 mM Tris-HCl buffer pH = 7.0, polyP (10 mM), AMP or ADP as substrates with a concentration from 0.5 to 2 mM, and MgSO_4_ (10 mM) as the metal ion cofactor. Reactions were initiated by the addition of *Bc*PPK2-III (20 µg/mL). The reaction rate was determined at early reaction times, ensuring that less than 10% of the substrate was converted. This approach guarantees that measurements were taken within the reaction’s “linear phase”, well before reaching equilibrium and at the initial step of the AMP→ADP or ADP→ATP reaction sequence.

Initial velocities (V, in U/mg) were fitted to the Hill equation, and kinetic constants (V_max_ and K_M_) were calculated using the built-in nonlinear regression tools in SigmaPlot 14.0 (Figure S8).

#### 3.4.4. Stability Assays of *Bc*PPK2-III

Thermostability and pH profile assays were performed with a final enzyme concentration of 10 µg/mL. The temperature gradient scale ranging from 30 to 70 °C was established as follows: 30.0, 31.6, 34.6, 39.5, 45.3, 49.6, 52.8, 55, 56.1, 57.9, 60.7, 64, 67, 68.9, and 70 °C. This gradient profile was achieved using a thermocycler (Bio-rad T100 Thermal Cycler). After 10 min of incubation, *Bc*PPK2-III samples were removed, chilled on ice for 10 min and incubated further at room temperature for 10 min. Finally, samples of 20 μL were added to 180 μL volume of reaction mixture containing 100 mM Tris-HCl buffer, pH = 7.0, polyP (10 mM), AMP (2 mM) and MgSO_4_ (10 mM).

The optimum pH activity was determined using 100mM Britton-Robinson buffer at different pH values (2.0, 3.0, 4.0, 5.0, 6.0, 7.0, 8.0, 9.0, 10.0, 11.0 and 12.0) containing the reaction mixture with the aforementioned concentrations of AMP, polyP and MgSO_4_.

### 3.5. Scanning Transmission Electron Microscopy

Transmission electron microscopy images were recorded with a field emission scanning electron microscope (FESEM), Hitachi SU-8000, operated at 30 kV in transmitted electron imaging mode (sTEM) equipped with a charge-coupled device (CCD) camera. Briefly, for sample preparation, one drop of reaction solution in water (~5–10 μL) was left to dry over ultrathin carbon type-A film supported on a 400-mesh copper grid (3 mm in diameter) (from Ted Pella, Inc. CA, USA).

## 4. Conclusions

It was experimentally demonstrated that the enzyme encoded by the *ppk2-III* gene is a polyphosphate kinase of family 2, subclass III, which is capable of producing ATP from AMP. Sequence analysis confirmed the presence of conserved motifs, including the Walker A and B motifs, characteristic of PPK2 enzymes. Structural characterization through 3D modeling, molecular docking and peptide mass fingerprinting verified the identity and integrity of the recombinant protein.

The biochemical characterization of *Bc*PPK2-III revealed that the enzyme is active with several divalent cations, exhibiting substantial activity with Mg^2+^, Mn^2+^ and Co^2+^ and a distinct ADP production preference with Ca^2+^. The enzyme achieved a 70% conversion of ATP from AMP after 24 h, with a total turnover number of 4355. The reaction mechanism was determined to proceed through a stepwise phosphorylation process, forming ADP as an intermediate before generating ATP. In addition, the enzyme could be producing AP4 as a final product, when the first substrate of the reaction is ADP. According to the determined kinetic constants, the phosphorylation of AMP to ADP is the most efficient step.

Enzyme stability tests showed that *Bc*PPK2-III retains full activity at temperatures between 30 and 58 °C, with a T50 of 62 °C, indicating high thermostability. The enzyme also maintained more than 97% of its activity across a pH range of 6.0–9.0, with optimal activity at pH 9.0. These two properties are very valuable from a biocatalysis point of view, as in many processes—such as ATP regeneration—it is necessary to combine several enzymes with different activity optima or to work under very demanding conditions in order to adapt the process to an industrial scale.

These findings suggest that *Bc*PPK2-III is a robust and versatile enzyme with significant potential for use in ATP regeneration, reducing costs and improving the efficiency of biocatalytic processes that require stoichiometric amounts of ATP. The enzyme’s ability to function with multiple metal cofactors and its stability under various conditions make it a valuable candidate for industrial applications.

## Figures and Tables

**Table 1:** Molecular docking results.

Compound	Interacting Residues	Docking Score (kcal/mol)	Glide Emodel
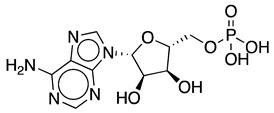	Thr78, Lys81, Gly80, Asp82, Arg133, Arg193, Lys202, Asp207	−7.0	−100.3
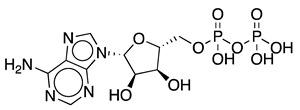	Gly80, Asp82, Arg133, Arg193, Lys202	−6.5	−110.3
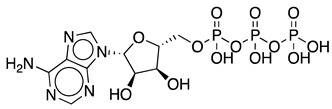	Gly80, Lys81, Asp82, Gly83, Lys 103, Arg133, Arg193, Lys202, Lys243	−6.3	−136.7

**Table 2:** Kinetic parameters of *Bc*PPK2-III.

Reaction	V_max_ (U/mg)	k_cat_ (s^−1^)	K_M_ (mM)	k_cat_/K_M_ (M^−1^s^−1^)	R^2^	ITR *	TTN ^§^
AMP to ATP	0.224 ± 0.011	0.119 ± 0.011	0.79 ± 0.04	151 ± 16	0.95	7.700 ± 0.001	4355
AMP to ADP	1.12 ± 0.034	0.594 ± 0.033	1.14 ± 0.33	520 ± 150	0.89	25.217 ± 0.001	
ADP to ATP	1.65 ± 0.15	0.88 ± 0.15	0.71 ± 0.06	1240 ± 230	0.96		

* µmol ATP µmol *Bc*PPK2-III^−1^min^−1^. § µmol ATP µmol *Bc*PPK2-III^−1^.

## Data Availability

The data that support the findings of this study are available from the corresponding authors upon reasonable request.

## Supplementary Materials

The following supporting information can be downloaded at: https://www.mdpi.com/article/10.3390/ijms252312995/s1.
