# Supplementary material for: Polyphosphate Kinase from Burkholderia cenocepacia, One Enzyme Catalyzing a Two-Step Cascade Reaction to Synthesize ATP from AMP
Source: Int J Mol Sci. 2024 Dec 3;25(23):12995. doi: 10.3390/ijms252312995 (PMC11641546; doi:10.3390/ijms252312995)
Supplement: Supplementary file 1 [file ijms-25-12995-s001.zip › ijms-3326116-supplementary.pdf]

Supporting Information

Polyphosphate Kinase from *Burkholderia cenocepacia*, One Enzyme Catalyzing a Two Step Cascade Reaction to Synthesize ATP from AMP

Dianelis T. Monterrey *,†, Leire Azcona, Julia Revuelta, Israel Sánchez-Moreno and Eduardo García-Junceda *

Department of Bio-Organic Chemistry, Instituto de Química Orgánica General, CSIC (IQOG-CSIC), Juan de la Cierva 3, 28006 Madrid, Spain; leireazcona.sa@gmail.com (L.A.); julia.revuelta@iqog.csic.es (J.R.); israel.sanchez@csic.es (I.S.-M.)

* Correspondence: d.monterrey@csic.es (D.T.M.); eduardo.junceda@csic.es (E.G.-J.)

† Current address: Department of Biocatalysis, ICP-CSIC, Marie Curie 2, 28049 Madrid, Spain.

Score	Expect	Method	Identities	Positives	Gaps
271 bits(692)	1e-95	Compositional matrix adjust	129/255(51%)	172/255(67%)	0/255(0%)

```
B. cepacia   24 FKLDAFDPAAKPFSSGSKEADRERLSVVSTELDVQQERLHTQQKKRVLLVLQGMDTSGKDGTVRA   88
M. ruber     25 FELKRFDPDDTSAFEGGKQAALEALAVLNRRLEKLQELLYAEGQHKVLVVLQAMDAGGKDGTIRV   89
                F+L  FDP       G K+A  E L+V++  L+  QE L+ + + +VL+VLQ MD  GKD GT+R

B. cepacia   89 VFREVDPLGLRIVPFKAPTPIEAAHDFLWRVHAQVPAAGELAIFNRSHYEDVLVPRVLGAIGDKE  153
M. ruber     90 VFDGVNPSGVRVASFGVPTEQELARDYLWRVHQQVPRKGELVIFNRSHYEDVLVVRVKNLVPQQV  154
                VF  V+P G+R+  F  PT  E A D+LWRVH QVP  GEL IFNRSHYEDVLV RV   +  +

B. cepacia  154 CERRYRQIRDFETMLFENGTTIIKCFLHISKDEQRARLQARIDDPTKHWKFDIADLDARKHWDAY  218
M. ruber    155 WQKRYRHIREFERMLADEGTTILKFFLHISKDEQRQRLQERLDNPEKRWKFRMGDLEDRRLWDRY  219
                 ++RYR IR+FE ML + GTTI+K FLHISKDEQR RLQ R+D+P K WKF + DL+ R+ WD Y

B. cepacia  219 QSAYRDALAATSAEHAPWYVIPANSKTHRNVMIAELLLRTMTDMKLEFPPPKPELEGVKI  278
M. ruber    220 QEAYEAAIRETSTEYAPWYVIPANKNWYRNWLVSHILVETLEGLAMQYPQPETASEKIVI  279
                Q AY  A+  TS E+APWYVIPAN   +RN +++ +L+ T+  + +++P P+   E + I
```

Figure S1. Sequence alignment of PPK2-III proteins from *B. cenocepacia* and *M. ruber* (Mrub_2488). Walker A and B motifs highlighted in red and blue, respectively and the cap module highlighted in purple; the conserved residues, glutamic acid (E137) is highlighted in green and glutamine (Q74) in yellow.

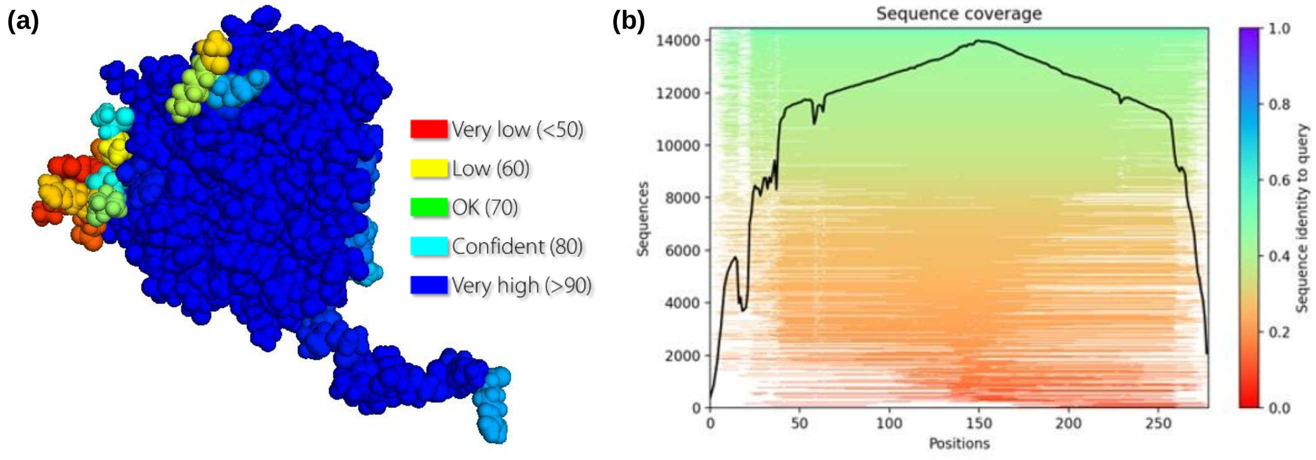

Figure S2. (**a**) AlphaFold2 prediction colored by model confidence band; (**b**) Sequence coverage. Number of homologous sequences identified per position

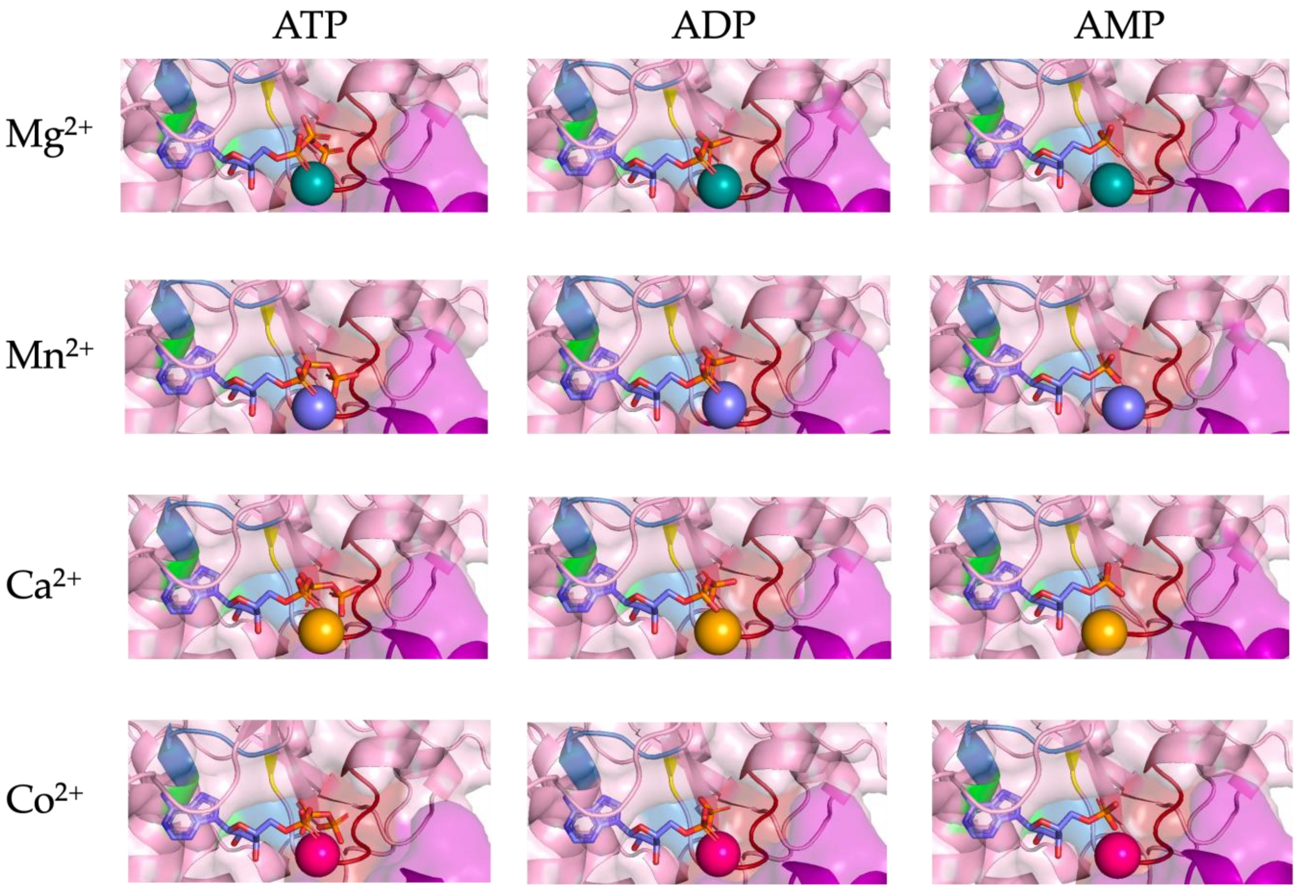

Figure S3. AlphaFold3 prediction of *Bc*PPK2-III bound to the corresponding divalent cation and ligands (ATP, ADP, or AMP). Key residues are highlighted: Walker A motif residues in red, Walker B motif residues in blue, glutamic acid (E) in green, glutamine (G) in yellow and the lid domain residues in violet.

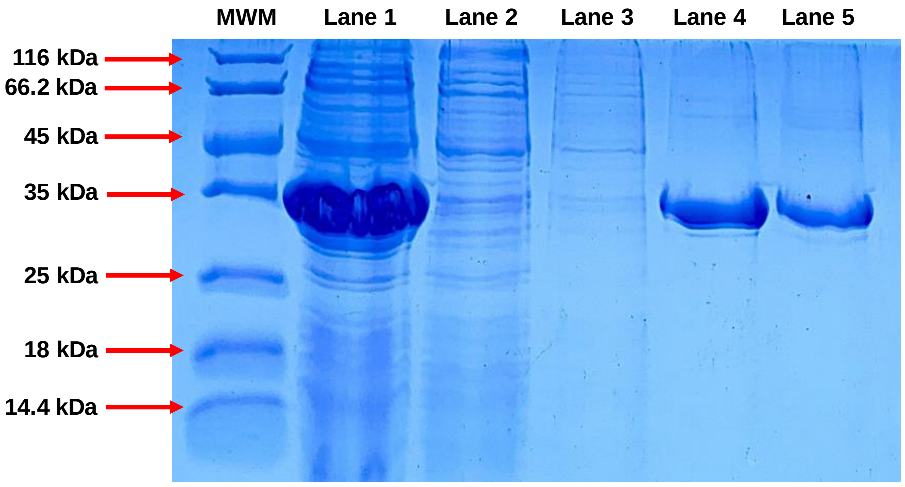

Figure S4. SDS-PAGE analysis of expression and IMAC purification of *Bc*PPK2-III. MWM: molecular weight marker; Lane 1: cell-free extract; Lane 2: pass-through fraction; Lane 3: elution of proteins nonspecifically bound to resin; Lane 4: elution of recombinant protein; Lane 5: resin with proteins not removed after washing and elution.

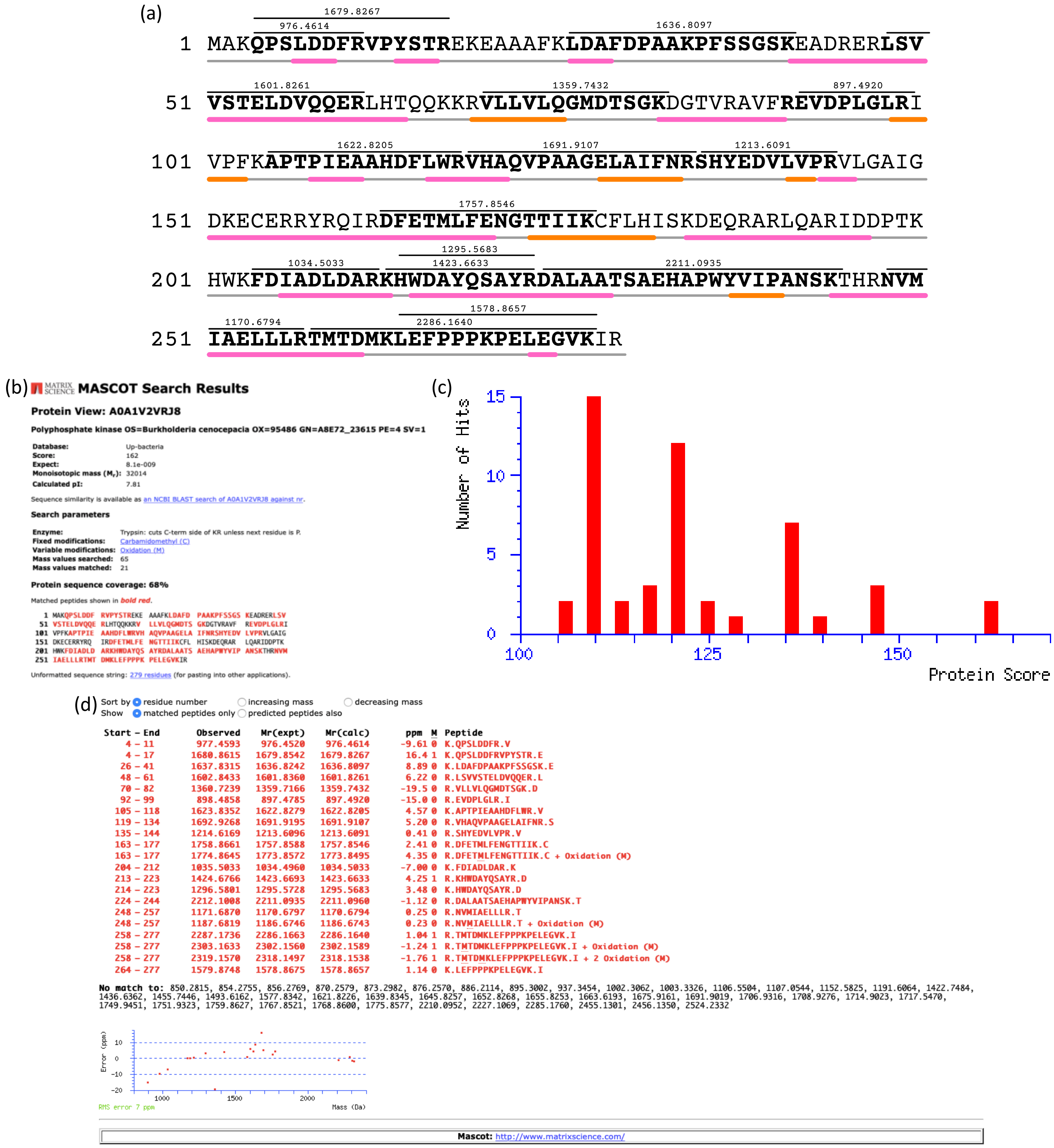

Figure S5. (a) Schematic representation of the peptide mass fingerprint and major secondary structural motifs of *Bc*PPK2-III. The identified peptides are highlighted in bold and underlined with their corresponding molecular mass in Dalton (Da). Sequences corresponding to α-helix are shown in pink and those corresponding to β-sheets in orange. Segments corresponding to "coil-coil" structures are highlighted in gray. (b), (c) and (d) raw data for the sequence reconstruction.

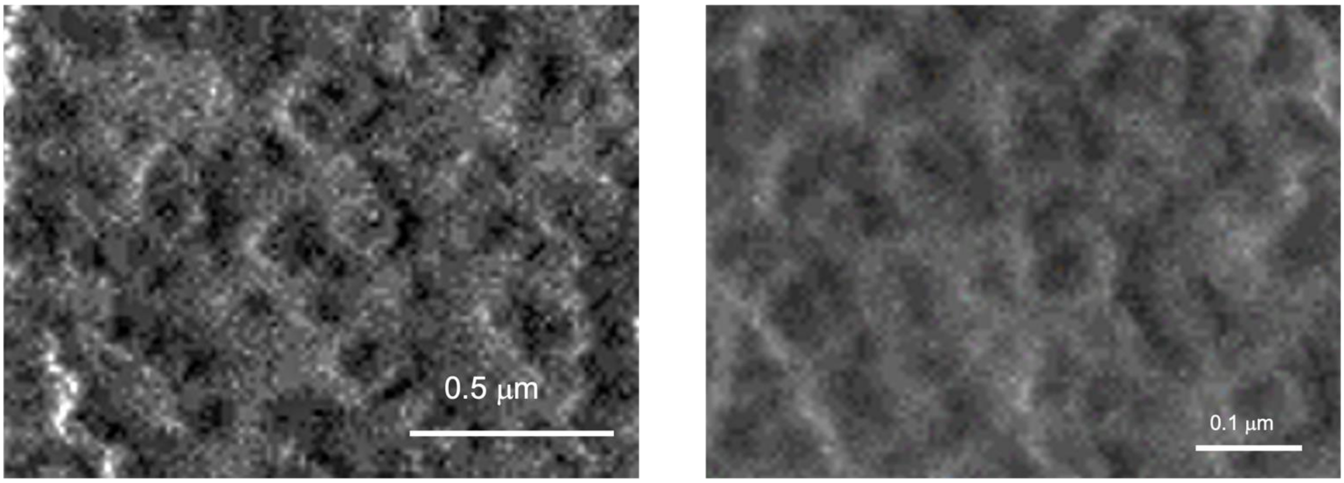

Figure S6. Transmission electron microscopy images (sTEM) of the nanoflower structure formed between Mn^{2+} and the shorter chains of phosphate donor groups.

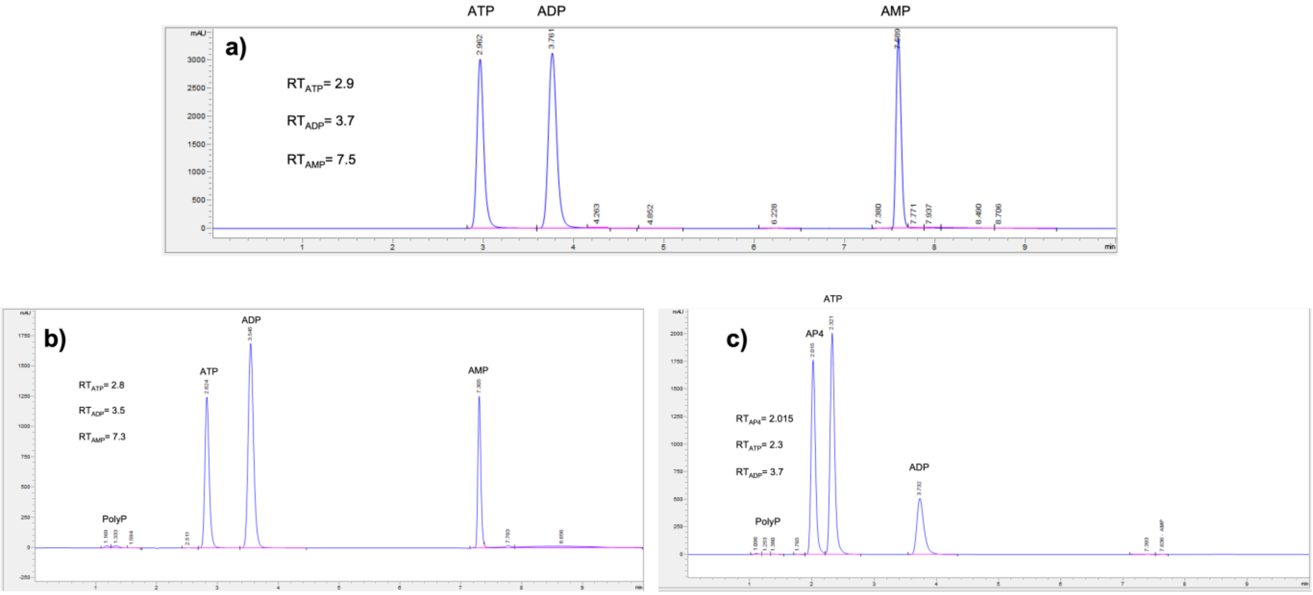

Figure S7. (**a**) Chromatogram of the standard mixture of polyP, ATP, ADP and AMP. (**b**) Chromatogram of *Bc*PPK2-III reaction after 3.5h, with AMP as the starting substrate. (**c**) Chromatogram of *Bc*PPK2-III reaction after 3.5h, with ADP as the starting substrate.

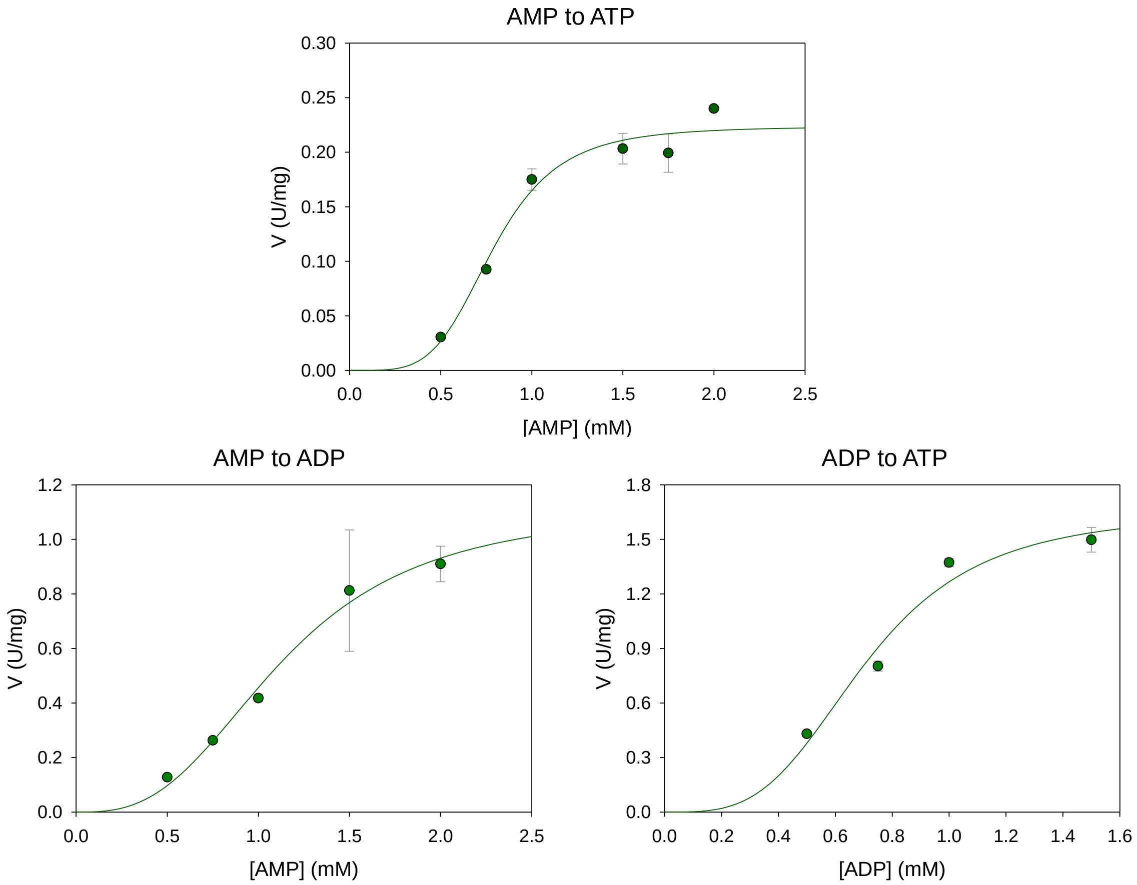

Figure S8. Hill equation fitting curves for enzyme kinetics of *Bc*PPK2-III. The graphs represent the nonlinear regression analysis of enzymatic activity as a function of substrate concentration, fitted to the Hill equation.

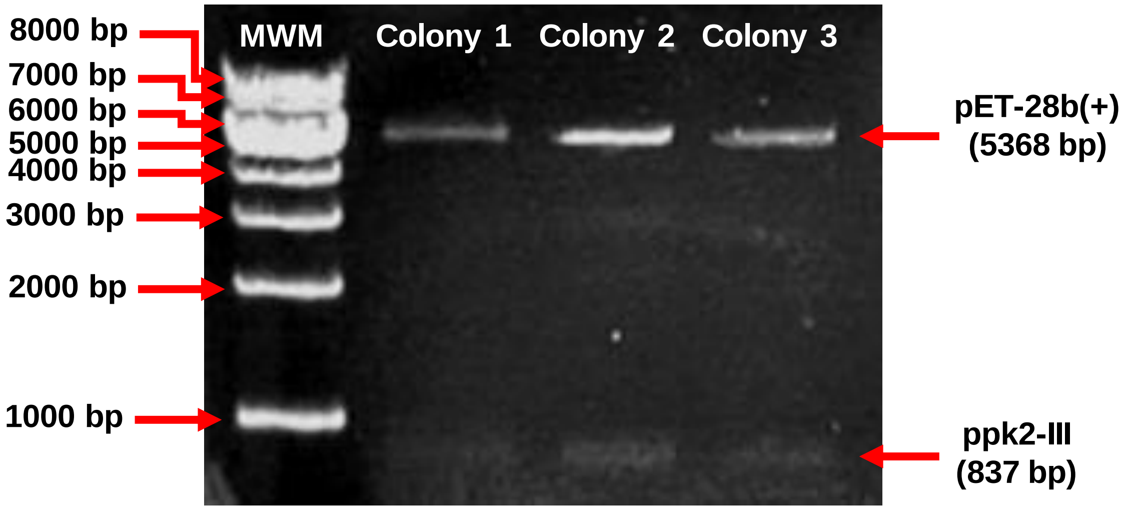

Figure S9. Agarose gel of restriction analysis of plasmid pET-28b(+)-*ppk-III*, purified from three different colonies. MWM = molecular weight marker.
